# Early targeted neonatal echocardiography identifies biventricular dysfunction and pulmonary hypertension associated with death or severe bronchopulmonary dysplasia in infants born before 29 weeks' gestation

**DOI:** 10.3389/fped.2026.1782985

**Published:** 2026-03-23

**Authors:** Abdulaziz Homedi, Kamal Ali, Faisal Alamer, Abdulrahman Mandurah, Mohammed Almahdi, Musab Alshareef, Saad Alshreedah, Faisal Alsehli, Tarek Mohamed, Ibrahim Ali

**Affiliations:** 1 Neonatal Intensive Care Department, Women’s Health Hospital, Ministry of National Guard Health Affairs, Riyadh, Saudi Arabia; 2 King Abdullah International Medical Research Center, Riyadh, Saudi Arabia; 3 King Saud Bin Abdulaziz University for Health Sciences, Riyadh, Saudi Arabia

**Keywords:** bronchopulmonary dysplasia, extreme prematurity, pulmonary hypertension, targeted neonatal echocardiography, ventricular dysfunction

## Abstract

**Background:**

Extremely preterm infants are at increased risk of haemodynamic instability during early postnatal transition. The contribution of early biventricular dysfunction and pulmonary hypertension to mortality and severe respiratory morbidity remains incompletely defined.

**Objectives:**

To determine whether early targeted neonatal echocardiography (TNE) markers of biventricular function and pulmonary hypertension are associated with death or severe bronchopulmonary dysplasia (BPD) in infants born before 29 weeks' gestation.

**Methods:**

We conducted a retrospective cohort study of inborn infants <29 weeks' gestation admitted to a tertiary neonatal intensive care unit between October 2023 and June 2025. All infants underwent standardized TNE within 72 h of birth. Echocardiographic indices of left and right ventricular systolic function, ductal shunt direction, and pulmonary hypertension were compared between survivors and non-survivors, and among survivors with and without severe BPD (Jensen grade 3). The primary outcome was death before 36 weeks' postmenstrual age or severe BPD.

**Results:**

Of 122 infants included, 24 (19.7%) died and 33 of 98 survivors (33.7%) developed severe BPD. Survivors demonstrated significantly better biventricular systolic performance and more favourable pulmonary haemodynamics, including higher ventricular outputs, greater tricuspid annular plane systolic excursion, higher pulmonary artery acceleration time–to–right ventricular ejection time ratios, and lower eccentricity indices (all *p* < 0.001). Non-survivors more frequently exhibited bidirectional or right-to-left ductal shunting, higher estimated right ventricular systolic pressure, and septal bowing. Among survivors, severe BPD was associated with worse right ventricular systolic function and higher pulmonary hypertension markers, while left ventricular systolic indices did not differ.

**Conclusions:**

In extremely preterm infants, early TNE markers of biventricular dysfunction and pulmonary hypertension were associated with death and severe respiratory morbidity. These findings suggest that early haemodynamic phenotype may have prognostic relevance and warrant further prospective investigation.

## Introduction

Extremely preterm infants are born with structurally and functionally immature myocardium, and abrupt postnatal changes in loading conditions can affect cardiomyocyte growth and extracellular matrix remodeling, contributing to early haemodynamic instability (1, 2). Transitional circulation in this population is further influenced by persistent fetal shunts, elevated pulmonary vascular resistance, evolving lung disease, and ventilator related effects, all of which may impair cardiac output during the early postnatal period (3–8). Together, these factors create a complex and highly dynamic haemodynamic environment in the immediate postnatal period.

Conventional measures of left ventricular systolic function, such as fraction shortening and ejection fraction, are widely used and feasible in neonates but are load dependent and may not fully capture early haemodynamic adaptation. Moreover, these indices primarily reflect left ventricular performance and do not comprehensively assess right ventricular function, particularly given the right ventricle's complex geometry. In contrast, deformation based measures, including speckle tracking strain, and standardized right sided indices such as tricuspid annular plane systolic excursion and fractional area change provide complementary information and may better characterize ventriculo pulmonary interactions in the early neonatal period (9–18).

Observational studies have reported associations between early right and left ventricular dysfunction, echocardiographic markers of acute pulmonary hypertension, and adverse outcomes in extremely preterm infants, including mortality and severe bronchopulmonary dysplasia (BPD) (19–27). However, much of the existing literature is constrained by selective rather than routine echocardiographic assessment, heterogeneous acquisition protocols, inconsistent definitions of haemodynamic impairment, and reliance on isolated single-parameter measurements. Consequently, population-level data derived from systematic early echocardiographic screening remain limited. Furthermore, as most of the available evidence is observational, causal inference is restricted and the overall strength of evidence remains moderate.

In our unit, a structured targeted neonatal echocardiography (TNE) service is routinely performed as part of clinical haemodynamic assessment in infants born at less than 29 weeks' gestation, with standardized evaluation within 72 h of birth. This study is a retrospective analysis of this established practice and examines whether early haemodynamic profiles identified during routine care are associated with clinically relevant outcomes.

The primary aim was to determine whether early TNE markers of biventricular dysfunction and acute pulmonary hypertension are associated with a composite outcome of death before 36 weeks' postmenstrual age or severe bronchopulmonary dysplasia (Jensen grade 3) (28). Secondary aims were to compare early TNE indices between outcome groups and to describe associated respiratory resource utilization.

## Materials and methods

### Study design and population

This was a retrospective cohort study conducted in the Neonatal Intensive Care Unit of Women's Health Hospital, Ministry of National Guard Health Affairs, Riyadh, Saudi Arabia. The study period extended from 1 October 2023 to 30 June 2025. Women's Health Hospital is a tertiary perinatal referral center with approximately 10,000 deliveries annually.

All inborn infants with gestational age below 29 completed weeks who were admitted to the NICU during the study period were eligible for inclusion. Infants with ductal dependent congenital heart disease or known chromosomal abnormalities were excluded. Clinical, demographic, and outcome data were extracted from the electronic medical record using standardized data collection forms.

### Outcomes

#### Primary outcome

The primary outcome was a composite of death before 36 weeks' postmenstrual age or severe bronchopulmonary dysplasia assessed at 36 weeks' postmenstrual age. Severe bronchopulmonary dysplasia was defined according to Jensen criteria as grade 3 respiratory support requirement at 36 weeks' postmenstrual age (28). No infants were discharged prior to 36 weeks' postmenstrual age.

#### Secondary outcomes

Secondary outcomes included the individual components of the primary outcome analyzed separately, namely death before 36 weeks' postmenstrual age and severe bronchopulmonary dysplasia. Additional secondary outcomes included survival without major morbidity, defined as survival to hospital discharge without severe intraventricular haemorrhage (Papile grade III or IV) (29), necrotising enterocolitis, or severe bronchopulmonary dysplasia.

Other prespecified clinical outcomes included duration of invasive mechanical ventilation (days), duration of supplemental oxygen therapy (days), neonatal intensive care unit length of stay (days), receipt of treatment for patent ductus arteriosus (pharmacologic and/or surgical), and retinopathy of prematurity requiring treatment.

#### Outcome ascertainment and follow up

All outcomes were ascertained from the electronic medical record using predefined operational definitions. Outcomes were assessed over the index hospital admission through 36 weeks' postmenstrual age or hospital discharge, whichever occurred first. Denominators are reported for each analysis.

#### Echocardiographic measurements

Echocardiographic examinations were performed using the Vivid ultrasound platform (GE HealthCare, Milwaukee, WI) with a neonatal phased-array multifrequency transducer (6–12 MHz). Studies were conducted by trained neonatologists with certification in targeted neonatal echocardiography, following standardized acquisition protocols consistent with published international recommendations (7). Standard imaging windows included subcostal, parasternal long axis, parasternal short axis, apical, ductal, and suprasternal views.

Assessment of haemodynamically significant patent ductus arteriosus was performed using the Iowa PDA echocardiographic scoring system (30). This structured score incorporates ductal diameter, shunt direction, indices of pulmonary overcirculation (including left atrial enlargement and increased left ventricular output), and markers of systemic hypoperfusion. Higher total scores reflect greater haemodynamic significance. The score was applied at the time of the early echocardiographic assessment as part of routine clinical evaluation (30).

Left ventricular systolic and diastolic function were evaluated using left ventricular output indexed to body weight, velocity time integral, ejection fraction, fractional shortening, and longitudinal strain derived from speckle tracking when image quality was adequate. Right ventricular assessment included right ventricular output indexed to body weight, velocity time integral, fractional area change, tricuspid annular plane systolic excursion, and right ventricular longitudinal strain.

Markers of acute pulmonary hypertension included estimation of systolic pulmonary arterial pressure derived from the tricuspid regurgitant jet when available, or from ductal flow direction with an assumed right atrial pressure of 5 mmHg. Additional indices included pulmonary artery acceleration time, right ventricular ejection time, the pulmonary artery acceleration time to ejection time ratio, and the left ventricular end-systolic eccentricity index as a quantitative measure of septal geometry. Interventricular septal configuration was also recorded qualitatively throughout systole (categorized as round, flat, or bowing) to describe overall septal morphology in conjunction with the quantitative eccentricity index.

Pulmonary overcirculation and systemic hypoperfusion were defined *a priori* according to predefined targeted neonatal echocardiography criteria. Pulmonary overcirculation was characterized by a hemodynamically significant left-to-right ductal shunt with echocardiographic evidence of increased pulmonary blood flow, including LA: Ao ratio ≥1.3 and elevated left ventricular output. Systemic hypoperfusion was defined by reduced systemic blood flow parameters, including low left ventricular output and reduced aortic velocity time integral, in conjunction with clinical signs of impaired perfusion.

#### Data sources and variables

Clinical, demographic, respiratory support, echocardiographic, and outcome data were extracted from the electronic medical record using standardized data collection forms and predefined variable definitions. Echocardiographic parameters were obtained from structured targeted neonatal echocardiography reports generated at the time of clinical assessment and recorded according to the institutional protocol. Clinical outcomes and resource utilization measures were abstracted from prospectively documented electronic records.

#### Power calculation

All eligible inborn infants born at less than 29 weeks' gestation during the study period were included using a consecutive sampling approach. As the targeted neonatal echocardiography pathway was implemented as a service wide clinical programme and the study represents an evaluation of routine practice, a formal *a priori* power calculation was not performed. The study period was selected to capture both the implementation and steady state phases of the programme while maintaining consistent imaging protocols and outcome definitions.

### Statistical analysis

Statistical analyses were performed to examine associations between early targeted neonatal echocardiography (TNE) findings, including markers of biventricular dysfunction and acute pulmonary hypertension, and clinical outcomes in infants born at less than 29 weeks' gestation. Categorical variables are presented as counts and percentages, and continuous variables as medians with interquartile ranges.

Comparisons between outcome groups were performed using the chi-square test or Fisher's exact test for categorical variables, as appropriate. Continuous variables were compared using the Mann–Whitney *U* test after assessment of distributional normality using the Shapiro–Wilk test, which confirmed non-normal distributions.

Multivariable associations between echocardiographic indices and adverse outcomes were examined using Firth penalized logistic regression to account for small event numbers and potential separation. Separate models were constructed for the primary composite outcome (death before 36 weeks' postmenstrual age or severe bronchopulmonary dysplasia), for death alone, and for bronchopulmonary dysplasia among survivors. Echocardiographic predictors were standardized per one standard deviation increase to allow comparison of effect sizes across parameters. Models were adjusted for gestational age, birth weight, sex, and antenatal steroid exposure. Given the physiological interdependence among echocardiographic parameters, predictors were entered into multivariable models individually rather than simultaneously to reduce potential multicollinearity and overfitting. Formal collinearity diagnostics were therefore not applicable to combined predictor models.

Discriminatory performance of selected echocardiographic indices for prediction of mortality was evaluated using receiver operating characteristic (ROC) curve analysis. Area under the curve (AUC) with 95% confidence intervals was calculated, and optimal cut-off values were determined using the Youden index. Sensitivity and specificity corresponding to these thresholds are reported.

All statistical tests were two-sided, and a *p* value less than 0.05 was considered statistically significant. Analyses were performed using IBM SPSS Statistics, version 31 (IBM Corp., Armonk, NY).

## Results

**Figure 1:**
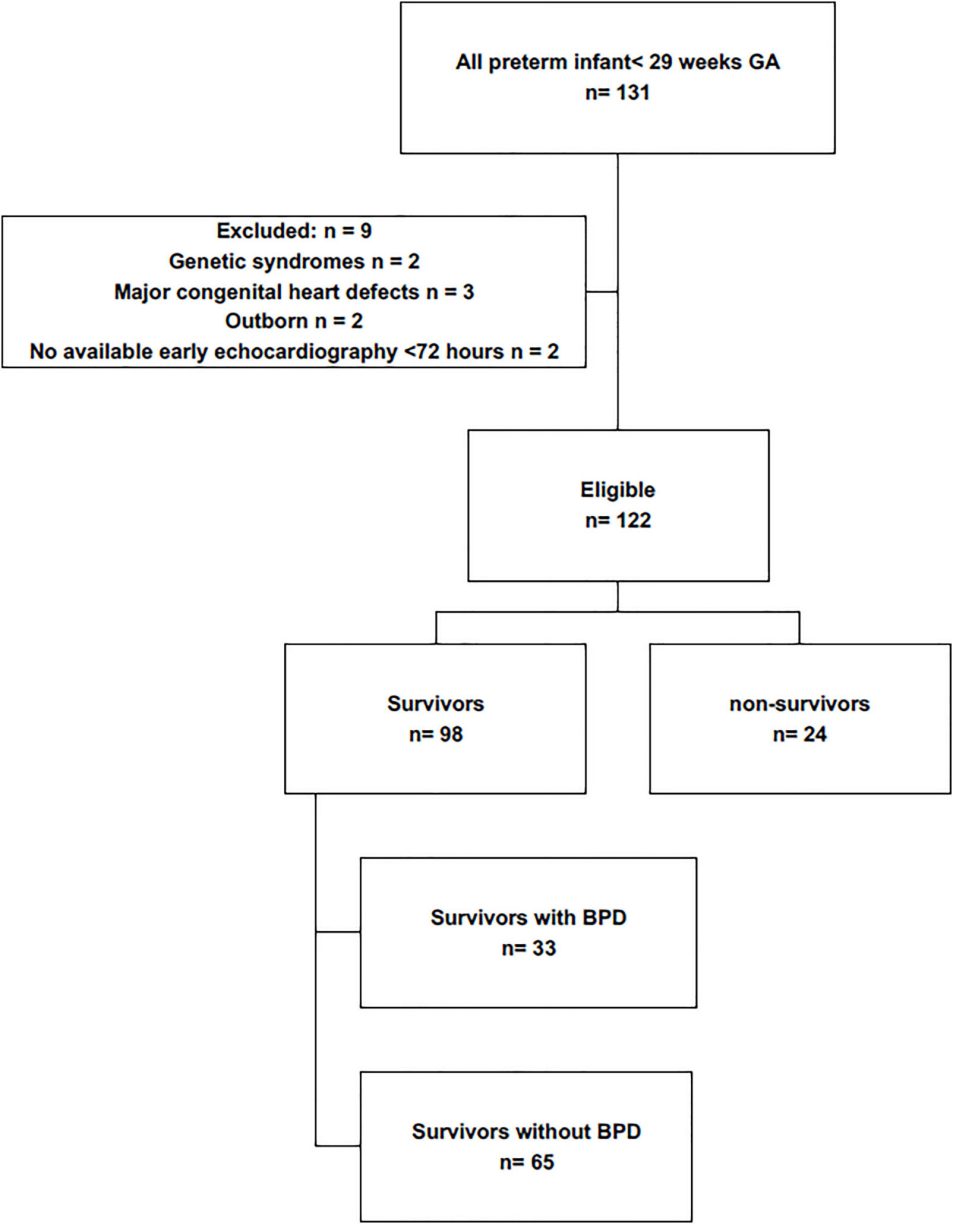
Study's flow diagram.

Of 131 inborn infants <29 weeks' gestational age screened, 9 were excluded (genetic/chromosomal syndromes, *n* = 2; major congenital heart defects, *n* = 3; outborn, *n* = 2; no early echocardiography <72 h, *n* = 2). The analytic cohort comprised 122 infants, of whom 98 survived and 24 died. Among survivors, 33 developed bronchopulmonary dysplasia (BPD) (Figure 1).

Table 1 presents baseline neonatal and maternal characteristics stratified by survival status. Survivors were born at a higher gestational age (median 27 vs. 25 weeks) and had higher birth weight (900 vs. 600 g) compared with non-survivors (both *p* < 0.001). Apgar scores at 5 min were also higher among survivors (median 8 vs. 6.5, *p* < 0.001). Caesarean delivery was more frequent in the survivor group. Length of stay was longer among survivors, consistent with early mortality in the non-survivor group. Other perinatal characteristics did not differ significantly between groups.

**Table 1:** Baseline neonatal and maternal characteristics by survival status.

Variable	Survivors (*n* = 98)	Non-survivors (*n* = 24)	*p* value
Gestational age, weeks	27 (26, 28)	25 (23, 26)	<0.001
Birth weight, g	900 (700, 1,100)	600 (540, 750)	<0.001
Male sex	57 (58.2)	16 (66.7)	0.446
Cesarean delivery	62 (63.3)	6 (25.0)	<0.001
Multiple pregnancy	27 (27.6)	7 (29.2)	0.874
Apgar score at 5 min	8 (7, 8)	6 (5, 7)	<0.001
Apgar score at 10 min	8 (7, 8)	7 (6, 8)	0.178
Antenatal steroids	88 (89.8)	21 (87.5)	0.705
Magnesium sulfate	87 (88.8)	18 (75.0)	0.087
Maternal chorioamnionitis	3 (3.1)	2 (8.3)	0.258
PDA treatment	39 (39.8)	1 (4.2)	<0.001
Age at PDA treatment start, days	2 (2, 3)	3 (3, 3)	0.550
Length of stay, days	82.5 (64, 112)	5 (1, 9)	<0.001

Data are presented as median (interquartile range) or *n* (%). Percentages are column-based. GA, gestational age; IQR, interquartile range; PDA, patent ductus arteriosus.

Table 2 compares echocardiographic parameters between survivors and non-survivors. Survivors demonstrated consistently superior biventricular systolic and diastolic performance. Left ventricular output, velocity-time integral, ejection fraction, fractional shortening, diastolic filling (E/A ratio), and longitudinal strain were all significantly higher in survivors (all *p* < 0.001). Right ventricular performance showed a parallel pattern, with greater right ventricular output, velocity-time integral, fractional area change, tricuspid annular plane systolic excursion, and more negative strain values among survivors (all *p* < 0.001).

**Table 2:** Echocardiographic parameters among survivors and non-survivors.

Variable	Survivors (*n* = 98)	Non-Survivors (*n* = 24)	*p* value
PDA size (mm)	1.70 (1.00, 2.00)	1.85 (1.30, 2.40)	0.133
Pulmonary over-circulation	39 (73.6%)	14 (26.4%)	0.101
Systemic hypoperfusion	39 (88.6%)	5 (11.4%)	0.083
IOWA score	6.0 (2.0, 7.0)	3.5 (3.0, 4.0)	0.099
LV output (mL/kg/min)	250 (210, 285)	93 (85, 110)	<0.001
LV VTI (Ao)	7.0 (5.9, 8.0)	2.8 (2.2, 3.4)	<0.001
LV E/A ratio	0.75 (0.60, 0.82)	0.33 (0.25, 0.50)	<0.001
LV ejection fraction (%)	59 (55, 63)	41 (39, 45)	<0.001
LV fractional shortening (%)	35 (35, 37)	23 (19, 28)	<0.001
LV STE (pLS, %)	−22 (−22, −21)	−16 (−18, −15)	<0.001
RV output (mL/kg/min)	310 (290, 335)	115 (100, 128)	<0.001
RV VTI (PA)	8.0 (6.9, 9.0)	3.3 (3.0, 4.0)	<0.001
RV FAC (%)	45 (45, 47)	26 (20, 33)	<0.001
RV TAPSE (cm)	0.6 (0.6, 0.7)	0.3 (0.3, 0.4)	<0.001
RV STE (pLS, %)	−20 (−21, −20)	−15 (−17, −13)	<0.001
PAAT/RVET	0.30 (0.29, 0.33)	0.19 (0.15, 0.23)	<0.001
Eccentricity index	1.1 (1.0, 1.2)	1.3 (1.3, 1.5)	<0.001

PDA, patent ductus arteriosus; hsPDA, hemodynamically significant PDA; LV, left ventricle; RV, right ventricle; VTI, velocity-time integral; STE, speckle-tracking echocardiography; pLS, peak longitudinal strain; FAC, fractional area change; TAPSE, tricuspid annular plane systolic excursion; PAAT/RVET, pulmonary artery acceleration time to right ventricular ejection time ratio; IOWA, Iowa PDA score. Values are median (interquartile range) or *n* (%).

Pulmonary haemodynamic indices also differed substantially between groups. Survivors had higher PAAT/RVET ratios and lower eccentricity indices, consistent with lower pulmonary vascular load. In contrast, non-survivors more frequently demonstrated septal bowing into the left ventricle and adverse ductal shunt patterns, including bidirectional or right-to-left shunting (*p* < 0.001). Markers of haemodynamically significant patent ductus arteriosus, including ductal size, pulmonary over-circulation, systemic hypoperfusion, and IOWA score, did not differ significantly between survivors and non-survivors.

Table 3 compares echocardiographic parameters among survivors with and without bronchopulmonary dysplasia. Left ventricular systolic and diastolic indices were similar between groups. In contrast, survivors who developed BPD demonstrated evidence of greater right ventricular and pulmonary vascular involvement, including lower right ventricular fractional area change, higher eccentricity index, higher estimated right ventricular systolic pressure, and more frequent abnormal septal configuration. Ductal shunt patterns differed significantly, with bidirectional shunting more common among infants with BPD. Markers of haemodynamically significant ductus arteriosus, including ductal size, IOWA score, and PAAT/RVET, were otherwise similar between groups.

**Table 3:** Echocardiographic parameters among survivors with and without bronchopulmonary dysplasia.

Variable	Survivors with BPD (*n* = 33)	Survivors without BPD (*n* = 65)	*p* value
PDA size (mm)	1.60 (1.00, 2.00)	1.70 (1.00, 2.00)	1.000
Pulmonary over-circulation	15 (45.5%)	24 (36.9%)	0.415
Systemic hypoperfusion	15 (45.5%)	24 (36.9%)	0.415
IOWA score	6.0 (2.0, 8.0)	6.0 (2.0, 7.0)	0.882
LV output (mL/kg/min)	250 (215, 290)	240 (210, 270)	0.500
LV VTI	7.0 (6.0, 8.0)	6.9 (5.9, 7.5)	0.668
LV E/A ratio	0.75 (0.60, 0.82)	0.75 (0.60, 0.81)	0.547
LV ejection fraction (%)	59 (55, 61)	59 (57, 64)	0.287
LV fractional shortening (%)	35 (34, 39)	36 (35, 37)	0.698
LV longitudinal strain (%)	−22 (−22, −20)	−21 (−22, −21)	0.802
RV output (mL/kg/min)	320 (300, 340)	300 (290, 320)	0.456
RV VTI	8.0 (6.9, 8.9)	8.0 (7.0, 9.0)	0.433
RV fractional area change (%)	45 (43, 45)	46 (45, 47)	0.009
RV TAPSE (cm)	0.6 (0.5, 0.7)	0.7 (0.6, 0.8)	0.082
RV longitudinal strain (%)	−20 (−21, −20)	−20 (−20, −20)	0.432
PDA shunt: Left-to-right	22 (66.7%)	40 (61.5%)	0.027
PDA shunt: Right-to-left	0 (0.0%)	0 (0.0%)	
PDA shunt: Bidirectional	7 (21.2%)	2 (3.1%)	
Estimated RV systolic pressure elevated	18 (54.5%)	19 (29.2%)	0.015
PAAT/RVET	0.30 (0.28, 0.33)	0.30 (0.29, 0.33)	0.442
Eccentricity index	1.2 (1.1, 1.2)	1.1 (1.0, 1.1)	<0.001
Septal morphology: Normal	15 (45.5%)	55 (84.6%)	<0.001
Septal morphology: Flat	17 (51.5%)	10 (15.4%)	
Septal morphology: Bowing	1 (3.0%)	0 (0.0%)	

BPD, bronchopulmonary dysplasia; PDA, patent ductus arteriosus; LV, left ventricle; RV, right ventricle; VTI, velocity-time integral; TAPSE, tricuspid annular plane systolic excursion; PAAT/RVET, pulmonary artery acceleration time to right ventricular ejection time ratio. Values are median (interquartile range) or *n* (%). PDA-related percentages are calculated among infants with a patent ductus arteriosus detected on early echocardiography and may not sum to the total survivor group (*n* = 98).

Table 4 summarizes neonatal morbidities and resource utilization among survivors stratified by the presence of BPD. Infants who developed BPD required substantially longer respiratory support, including prolonged invasive ventilation and oxygen therapy, and had a significantly longer NICU length of stay (all *p* < 0.001). Retinopathy of prematurity requiring treatment was also more frequent among infants with BPD. In contrast, rates of severe intraventricular hemorrhage, periventricular leukomalacia, necrotizing enterocolitis, and time to achieve full enteral feeds did not differ significantly between groups.

**Table 4:** Morbidities and resource use among survivors with and without BPD.

Variable	Survivors with BPD (*n* = 33)	Survivors without BPD (*n* = 65)	*p* value
Gestational age (weeks)	26 (25, 27)	27 (26, 28)	<0.001
Birthweight (grams)	800 (650, 900)	1,000 (787.5,1,100)	<0.001
IVH (grade III–IV)	4 (12.1)	3 (4.6)	0.427
IVH (grade I–II)	17 (51.5)	27 (41.5)	
Days on invasive ventilation	31.0 (11.0, 46.0)	8.0 (4.0, 18.0)	<0.001
Days on oxygen	97.0 (81.0, 117.0)	54.0 (44.0, 62.0)	<0.001
Days to full feeds	20.0 (13.0, 36.0)	16.0 (12.0, 25.0)	0.297
Periventricular leukomalacia	3 (9.1)	8 (12.3)	0.746
ROP requiring treatment	10 (30.3)	3 (4.6)	<0.001
Necrotizing enterocolitis	3 (9.1)	7 (10.8)	1.000
Length of stay (days)	109.0 (95.0, 128.0)	70.0 (58.0, 93.0)	<0.001

Values are median (interquartile range) or *n* (%). IVH, intraventricular hemorrhage.

Table 5 shows early echocardiographic indices according to the primary composite outcome. Infants who met the composite endpoint demonstrated lower left and right ventricular systolic performance and higher pulmonary pressure load compared with those without the composite outcome. Differences were observed across conventional systolic measures, myocardial deformation indices, and pulmonary haemodynamic parameters.

**Table 5:** Early echocardiographic indices according to the primary composite outcome.

Echocardiographic variable	Death or Severe BPD (*n* = 27)	No/Mild/Moderate BPD (*n* = 95)	*p* value
PDA size (mm)	1.80 (1.20, 2.32)	1.10 (0.00, 2.00)	0.005
PDA shunt pattern (1 = L → R, 2 = R → L, 3 = Bidirectional)	2.00 (1.00, 3.00)	1.00 (1.00, 1.00)	<0.001
Systemic circulation flow reversal (1 = Yes, 2 = No)	2.00 (2.00, 2.00)	2.00 (1.00, 2.00)	0.032
LV VTI (Ao)	2.90 (2.25, 3.50)	7.00 (6.00, 8.00)	<0.001
LVO (mL/kg/min)	100.00 (85.00, 112.50)	250.00 (210.00, 287.50)	<0.001
LVEF (%)	41.00 (39.00, 46.00)	59.00 (55.50, 63.00)	<0.001
LVFS (%)	25.00 (19.00, 28.00)	35.00 (35.00, 37.00)	<0.001
LV global strain (%)	−17.00 (−18.00, −15.00)	−22.00 (−22.00, −21.00)	<0.001
RV VTI (PA)	3.50 (3.00, 4.00)	8.00 (7.00, 9.00)	<0.001
RVO (mL/kg/min)	120.00 (102.50, 130.00)	310.00 (290.00, 335.00)	<0.001
TAPSE (cm)	0.30 (0.30, 0.40)	0.60 (0.60, 0.70)	<0.001
RVFAC (%)	29.00 (20.50, 36.00)	45.00 (45.00, 47.00)	<0.001
RV global strain (%)	−15.00 (−17.50, −13.00)	−20.00 (−21.00, −20.00)	<0.001
PAAT/RVET	0.19 (0.15, 0.27)	0.30 (0.29, 0.33)	<0.001
Eccentricity index	1.30 (1.25, 1.40)	1.10 (1.00, 1.20)	<0.001

BPD, bronchopulmonary dysplasia; PDA, patent ductus arteriosus; LV VTI, left ventricular velocity time integral; LVO, left ventricular output; LVEF, left ventricular ejection fraction; LVFS, left ventricular fractional shortening; RV VTI, right ventricular velocity time integral; RVO, right ventricular output; TAPSE, tricuspid annular plane systolic excursion; RVFAC, right ventricular fractional area change; PAAT/RVET, pulmonary artery acceleration time to right ventricular ejection time ratio.

Table 6 shows the adjusted associations between early echocardiographic indices and adverse outcomes using Firth penalized logistic regression. Several markers of ventricular dysfunction and pulmonary pressure loading were independently associated with the composite outcome of death or severe BPD. Associations were substantially stronger for mortality, whereas few parameters remained independently associated with BPD among survivors.

**Table 6:** Adjusted associations between early echocardiographic indices and adverse outcomes.

Echocardiographic predictor (per 1 SD increase)	Composite: death or BPD aOR (95% CI)	*p* value	Death aOR (95% CI)	*p* value	BPD among survivors aOR (95% CI)	*p* value
Eccentricity index	3.37 (1.79–6.34)	<0.001	5.23 (2.42–11.33)	<0.001	2.73 (1.26–5.91)	0.011
LV global strain	2.52 (1.37–4.66)	0.003	47.16 (6.69–332.42)	<0.001	1.03 (0.40–2.68)	0.951
LVEF	0.43 (0.25–0.75)	0.003	<0.01 (<0.01–0.08)	0.002	0.97 (0.44–2.11)	0.932
LVFS	0.39 (0.21–0.72)	0.003	0.02 (<0.01–0.14)	<0.001	1.10 (0.39–3.10)	0.852
LVO	0.50 (0.30–0.84)	0.008	0.04 (0.01–0.15)	<0.001	1.17 (0.56–2.43)	0.677
PAAT/RVET	0.56 (0.32–0.98)	0.041	0.09 (0.03–0.26)	<0.001	0.92 (0.56–1.50)	0.730
RV global strain	2.46 (1.30–4.65)	0.006	35.68 (5.75–221.40)	<0.001	0.75 (0.25–2.32)	0.622
RVFAC	0.15 (0.04–0.53)	0.003	0.01 (<0.01–0.14)	<0.001	0.23 (0.04–1.28)	0.093
RVO	0.47 (0.27–0.81)	0.007	0.05 (0.02–0.18)	<0.001	2.25 (0.67–7.55)	0.188
TAPSE	0.48 (0.28–0.82)	0.007	0.01 (<0.01–0.11)	<0.001	0.97 (0.49–1.93)	0.931

BPD, bronchopulmonary dysplasia; LVEF, left ventricular ejection fraction; LVFS, left ventricular fractional shortening; LVO, left ventricular output; PAAT/RVET, pulmonary artery acceleration time to right ventricular ejection time ratio; RVFAC, right ventricular fractional area change; RVO, right ventricular output; TAPSE, tricuspid annular plane systolic excursion.

Table 7 shows the receiver operating characteristic (ROC) analysis of early echocardiographic indices for prediction of mortality before discharge. Left and right ventricular systolic parameters demonstrated high discriminatory performance, with LVEF, LVFS, LVO, RVO, TAPSE, and RVFAC showing excellent AUC values (all *p* < 0.001). Myocardial deformation parameters and the eccentricity index were also significantly associated with mortality.

**Table 7:** Receiver operating characteristic (ROC) analysis for prediction of mortality before discharge.

Echocardiographic index	AUC (95% CI)	*p* value	Optimal cut-off	Sensitivity (%)	Specificity (%)
LVO (mL/kg/min)	0.967 (0.904–1.00)	<0.001	≤135	100	95.8
LVEF (%)	0.999 (0.996–1.00)	<0.001	≤52	96.9	100
LVFS (%)	0.992 (0.980–1.00)	<0.001	≤31.5	93.8	100
RVO (mL/kg/min)	0.973 (0.922–1.00)	<0.001	≤141	100	95.8
TAPSE (cm)	0.984 (0.966–1.00)	<0.001	≤0.45	94.8	91.7
RVFAC (%)	0.983 (0.951–1.00)	<0.001	≤40.5	95.9	95.8
LV global strain (%)	0.986 (0.972–1.00)	<0.001	≥−19.5	100	92.8
RV global strain (%)	0.979 (0.958–1.00)	<0.001	≥−19.5	100	81.4
Eccentricity index	0.875 (0.774–0.975)	<0.001	≥1.25	79.2	92.8

LVO, left ventricular output; LVEF, left ventricular ejection fraction; LVFS, left ventricular fractional shortening; RVO, right ventricular output; TAPSE, tricuspid annular plane systolic excursion; RVFAC, right ventricular fractional area change.

## Discussion

This single centre retrospective cohort study evaluated whether routine early targeted neonatal echocardiography in infants born at less than 29 weeks' gestation identifies haemodynamic features associated with death before 36 weeks' postmenstrual age or severe bronchopulmonary dysplasia. Early echocardiographic assessment within the first 72 h of life demonstrated significant differences in ventricular function and pulmonary haemodynamics between outcome groups. Both left and right ventricular performance, together with markers of pulmonary pressure loading and septal geometry, were associated with survival, supporting the potential value of systematic early haemodynamic screening in this population.

Early targeted neonatal echocardiography parameters differed according to survival status. Survivors demonstrated higher left and right ventricular outputs, higher left ventricular ejection fraction and fractional shortening, more favourable longitudinal strain values, and higher left ventricular E/A ratios, consistent with better global systolic and diastolic function. Pulmonary haemodynamics were also more favourable among survivors, with predominantly left to right ductal shunting, higher PAAT to RVET ratios, lower eccentricity indices, and less frequent septal flattening or bowing. In contrast, ductal size and composite haemodynamically significant PDA scores did not differ between groups, suggesting that ventricular performance and pulmonary vascular load, rather than ductal calibre alone, were more closely related to adverse outcome.

Among survivors, the haemodynamic pattern associated with subsequent bronchopulmonary dysplasia was primarily confined to right sided function and pulmonary pressure related measures. Left ventricular systolic and diastolic indices were similar in infants with and without bronchopulmonary dysplasia. However, infants who later developed bronchopulmonary dysplasia demonstrated lower right ventricular fractional area change, more frequent bidirectional ductal shunting, higher estimated right ventricular systolic pressure, higher eccentricity indices, and more abnormal septal configuration in the early postnatal period. These findings suggest that early right ventricular load and pulmonary vascular burden may be more closely linked to later bronchopulmonary dysplasia than left ventricular performance within a structured early echocardiography screening pathway.

Early echocardiographic assessment has been linked to later respiratory outcomes in extremely preterm infants. In a multicenter prospective cohort, Mourani et al. demonstrated that echocardiographic evidence of elevated pulmonary pressure and impaired ventricular performance in the early postnatal period were associated with subsequent bronchopulmonary dysplasia and pulmonary hypertension related to bronchopulmonary dysplasia, supporting the concept that early haemodynamic abnormalities identify infants at risk of downstream morbidity (31). Our findings are consistent with this trajectory, as indices of left and right ventricular performance and pulmonary hypertension were associated with differences in outcome in the early postnatal period. In preterm infants with respiratory failure, lung ultrasound scores positively correlated with both end-systolic and end-diastolic left ventricular eccentricity indices over the first 72 h of life. This suggests that worsening lung aeration may be associated with increased ventricular geometric distortion in early respiratory disease (32). The increased right ventricular afterload in early life promotes interventricular dependence, septal flattening, and elevation of tricuspid regurgitation–derived pressure estimates, mechanisms well described in pediatric pulmonary hypertension statements and evident in infants with adverse outcomes in our cohort (33). Contemporary reviews of haemodynamic assessment in preterm infants with chronic lung disease similarly emphasize Doppler based estimates of right ventricular pressure, septal geometry, and ductal flow direction as practical and clinically relevant markers, aligning closely with our findings (34).

The specific echocardiographic metrics used in this study are supported by pediatric reference data and guideline recommendations. Tricuspid annular plane systolic excursion has established age dependent reference values in infants and children and is recommended as a simple and reproducible measure of right ventricular systolic function; lower values are associated with impaired right ventricular performance, consistent with the differences observed between groups in our study (35). For assessment of pulmonary vascular load, the pulmonary artery acceleration time to right ventricular ejection time ratio is a feasible surrogate in neonates. Shorter acceleration time and lower ratios have been associated with higher pulmonary arterial pressure and worse outcomes in preterm cohorts, findings that are consistent with our observations (33).

Previous studies using targeted neonatal echocardiography have shown that measures of biventricular function and pulmonary vascular load obtained in the first days of life are associated with later respiratory outcomes in extremely preterm infants. Our results align with this literature, as infants who died or developed severe bronchopulmonary dysplasia demonstrated lower left and right ventricular systolic indices together with multiple markers consistent with higher pulmonary arterial pressure. Similar associations between early ventricular dysfunction, elevated pulmonary pressure, and the composite outcome of death or severe bronchopulmonary dysplasia have been reported in other cohorts, supporting the clinical value of early structured haemodynamic assessment (36, 37).

In addition to group differences, multivariable modelling demonstrated that several early echocardiographic parameters remained independently associated with adverse outcomes after adjustment for gestational age, birth weight, sex, and antenatal steroid exposure. Markers of ventricular dysfunction and pulmonary pressure loading were strongly associated with the composite outcome, with larger effect sizes observed for mortality than for bronchopulmonary dysplasia among survivors. Although Firth penalized regression was used to mitigate small-sample bias, the limited number of mortality events resulted in wide confidence intervals for some predictors, indicating reduced estimate precision and the need for cautious interpretation. Receiver operating characteristic analysis demonstrated excellent discriminatory performance of multiple systolic indices for mortality within this cohort. However, in the absence of internal validation techniques such as bootstrapping and given the limited event count, these AUC estimates may be optimistic and require confirmation in larger prospective cohorts before being considered robust prognostic tools. Moreover, the strong intercorrelation among systolic parameters suggests that the observed performance likely reflects pronounced haemodynamic separation between groups within this dataset rather than definitive predictive thresholds.

Several pulmonary hypertension–related findings in our study, including higher estimated right ventricular systolic pressure derived from tricuspid regurgitation, abnormal septal geometry reflected by an increased end-systolic eccentricity index, and lower pulmonary artery acceleration time to right ventricular ejection time ratios, are consistent with accepted non-invasive surrogates of elevated pulmonary vascular resistance in neonates. Pediatric pulmonary hypertension statements and neonatal reviews recommend integrating these parameters rather than relying on a single metric, acknowledging their individual limitations. Our data support this approach, as associations were most evident when pressure estimates, septal configuration, ductal flow characteristics, and doppler timing indices were interpreted together (36).

Our findings also highlight the role of the right ventricle in preterm respiratory morbidity. Prior studies have shown that right ventricular systolic indices such as tricuspid annular plane systolic excursion and fractional area change correlate with disease severity in bronchopulmonary dysplasia and adverse outcomes. In our cohort, lower right ventricular fractional area change, and tricuspid annular plane systolic excursion were observed in non-survivors and in survivors who later developed bronchopulmonary dysplasia, supporting the concept that right ventricular–pulmonary vascular coupling may be an important determinant of clinical course (25). In contrast, although left ventricular systolic indices differentiated survival status, they did not distinguish bronchopulmonary dysplasia among survivors, suggesting a closer association between pulmonary vascular load, right ventricular performance, and chronic lung disease.

Finally, our findings are consistent with neonatal haemodynamics programs that advocate for standardized early targeted neonatal echocardiography within a multiparameter framework. The protocol used in this study aligns with published neonatal haemodynamic guidance and pediatric echocardiography statements emphasizing integrated assessment of ductal haemodynamics, right ventricular afterload, septal geometry, and biventricular function. Such an approach may facilitate early identification of high-risk haemodynamic profiles in extremely preterm infants (38).

This study has several important strengths. It was conducted within a standardized early TNE program with predefined acquisition protocols and systematic assessment within the first 72 h of life, minimizing variability in image acquisition and interpretation. Comprehensive evaluation of both left and right ventricular systolic function, myocardial deformation, septal geometry, ductal haemodynamics, and Doppler timing indices allowed for an integrated multiparameter haemodynamic assessment rather than reliance on isolated measures. Inclusion of all eligible inborn infants reduces referral and ascertainment bias and enhances internal validity. Furthermore, multivariable modelling using Firth penalized logistic regression was applied to account for small event numbers and reduce separation bias, strengthening the robustness of adjusted associations.

Despite these strengths, several limitations should be considered. This was a single-centre retrospective analysis, which may limit generalisability and introduces potential selection and information bias. Infants who did not survive were of lower gestational age and birth weight and had markers of greater perinatal illness severity, which may partly account for observed differences in echocardiographic parameters. Although multivariable adjustment was performed, residual confounding inherent to observational studies cannot be excluded; therefore, findings should be interpreted as associative rather than indicative of independent predictive or causal effects. Echocardiographic assessment was performed at a single early time point, which does not capture the temporal evolution of ventricular function or pulmonary vascular load, and the absence of serial data limits evaluation of dynamic haemodynamic changes. In addition, multiple echocardiographic variables were compared across groups, introducing a potential risk of type I error. Finally, because echocardiography was performed as part of routine clinical care, findings may have influenced management decisions, including vasoactive support or PDA-directed treatment, which could have affected outcomes and represent potential treatment-related confounding.

## Conclusion

In infants born before 29 weeks' gestation, early standardized targeted neonatal echocardiography performed within 72 h identified distinct haemodynamic profiles associated with death before 36 weeks' postmenstrual age or severe bronchopulmonary dysplasia. Biventricular systolic dysfunction and markers of increased pulmonary vascular load were independently associated with adverse outcomes, with the strongest associations observed for mortality. Among survivors, later bronchopulmonary dysplasia was primarily linked to right ventricular and pulmonary pressure–related indices rather than left ventricular systolic function.

These findings suggest that structured early haemodynamic assessment may help identify infants with adverse physiological trajectories in the immediate postnatal period. Prospective multicenter studies are required to validate these associations and to determine whether integration of early echocardiographic findings into clinical decision-making can improve outcomes.

## Data Availability

The raw data supporting the conclusions of this article will be made available by the authors, without undue reservation.
